# Application of unmanned aerial system structure from motion point cloud detected tree heights and stem diameters to model missing stem diameters

**DOI:** 10.1016/j.mex.2022.101729

**Published:** 2022-05-13

**Authors:** Neal C. Swayze, Wade T. Tinkham

**Affiliations:** Department of Forest and Rangeland Stewardship, Colorado State University

**Keywords:** Tree Extraction, Variable Window, Circle Fitting, Unmanned Aerial System, Forest Inventory and Analysis, Stand Monitoring

## Abstract

Monitoring of tree spatial arrangement is increasingly essential for restoration of dry conifer forests. The presented method was developed for high-density point clouds, like those from unmanned aerial system imagery, to extract and model individual tree location, height, and diameter at breast height (DBH). Extraction of tree locations and heights uses a variable window function searching point cloud-derived canopy height models. Tree DBH is extracted for a subset of point cloud trees using a slice at 1.32-1.42 m and a least-squares circle fitting algorithm. Extracted heights and DBHs are spatially matched and filtered against each tree's expected DBH predicted using a regional National Forest Inventory height to DBH relationship. Values remaining after filtering are used to create a site-specific height to DBH relationship for predicting missing DBH values. Applying the method in a ponderosa pine-dominated forest found that extracted height values exceeded the precision of field height measurement approaches, while the accuracy of extracted and modeled DBH values had a mean error of 0.79 cm.

• Leveraging National Forest Inventory to filter DBH values eliminates the need for *in situ* observations.
• Produces tree list for all extractable stems in the point cloud.
• Transferable to high-density point clouds in open-canopy forests.

## Graphical abstract

**Figure:**
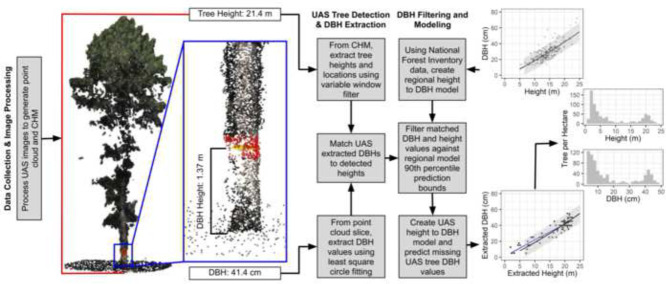

Specifications table

**Table:** 

Subject Area:	Agricultural and Biological Sciences
More specific subject area:	*Remote Sensing Augmented Forest Inventory*
Method name:	*Point Cloud-Based Estimation and Modeling of Tree Height and DBH Distributions*
Name and reference of original method:	*N.C. Swayze, W.T. Tinkham, J.C. Vogeler, A.T. Hudak. 2021. Influence of flight parameters on UAS-based monitoring of tree height, diameter, and density. Remote Sensing of Environment, in review.*
Resource availability:	*Supplement files of example datasets and processing scripts are provided.*

## Background

The demand for spatially explicit observations of tree heights and diameters at breast height (DBH; 1.37 m) has been increasing for assessment of forest management objectives [1], [2], [3] related to the spatial arrangement of trees and projecting tree-level growth [4]. However, extrapolation of height and DBH distributions from traditional field sampling lacks the information to inform spatial tree arrangements [5]. Additionally, the cost of stem-mapping individual trees across forest stands [6], [7] prohibits its implementation in management. To overcome these limitations, researchers have turned to remote sensing practices that provide 3-dimensional information related to forest structure [8], [9].

For over three decades, airborne laser scanning (ALS) has been the prominent technology for acquiring data points describing the vertical and horizontal heterogeneity of forest structure [10], [11]. As ALS data density and reliability have improved, more sophisticated analytical methods have been developed to detect individual tree location and heights [12], [13]. Local allometries can then be used to model tree DBH and biomass from these ALS-derived tree observations [14]. However, high ALS equipment costs and aircraft fees have drastically limited ALS use for the repeated acquisitions necessary for stand-level monitoring [15]. Although not designed for landscape-level monitoring like ALS, unmanned aerial systems (UAS) provide a scalable data acquisition strategy well suited for stand-level monitoring [8,16].

The integration of Structure from Motion (SfM) photogrammetry with UAS acquired high-resolution imagery can produce data densities exceeding 1,000 points m^−2^ [17]. These high data densities enable the application of ALS tree-level detection methods at a resolution capable of detecting >90% of overstory trees in open canopy conifer forests [16]. Additionally, when a UAS image acquisition strategy is utilized that facilitates image view angles that see lower into the forest canopy, detecting at least a subset of tree DBH values is feasible [18], [19]. Although not a complete list of DBH values, other UAS studies have shown that local allometries can predict missing tree observations [20], [21]. This paper presents a method for processing point clouds, such as those derived from UAS-SfM, to extract individual tree locations, heights, and DBHs along with a modeling routine for filling missing DBH observations without the need for *in situ* tree observation.

## Materials and methods

### Study area description

Development of the method occurred in a ponderosa pine (*Pinus ponderosa* Lawson & C. Lawson) dominated site at the Manitou Experimental Forest in the Pike-San Isabel National Forest of Colorado, USA. The forests at the site are an all-age ponderosa pine stand, characterized by a mosaic of spatially aggregated tree groups where each group generally contains a single cohort of tree sizes [22]. These forest structures developed after management in the 1880s that resembled a shelterwood harvest, followed by a century of regeneration pulses. Tree recruitment predominantly occurred in open areas because of ponderosa pine's shade intolerance [6].

### Data collection and image processing

**Fig. 1:**
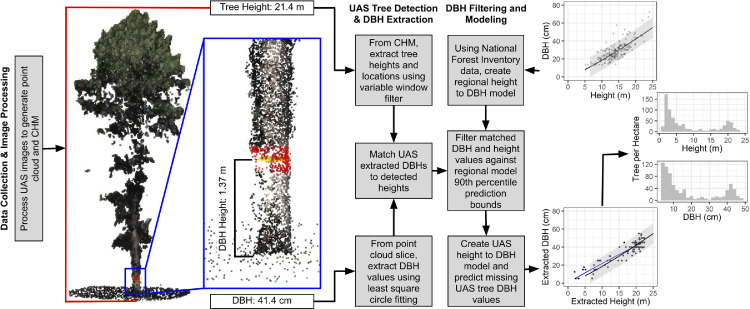
Workflow diagram of tree metric extraction, spatial matching, regional filtering, and UAS prediction (adapted from Swayze et al. [18]).

This method uses three stages, including 1) data collection and image processing, 2) tree height and DBH extraction, and 3) DBH filtering and modeling (Fig. 1). The method was initially tested against 30 UAS acquisitions at various altitudes, flight patterns, and camera angles by Swayze et al. [18] using a Phantom 4 Pro UAS over a 1-hectare study area, averaging 243 red-green-blue 20-megapixel images from each acquisition. Processing the imagery in Agisoft Metashape 1.6.4 generated dense SfM point clouds, following Tinkham & Swayze [17]. The resulting point clouds had point densities ranging from 916 to 6,086 points m^−2^, with varying levels of detail for understory vegetation and stem reconstruction. High-density point clouds in similar open-canopy forest systems should be suitable for this method. However, point clouds of forest systems with higher canopy cover or substantially reduced point densities will likely have less detail in the understory and stem regions. Such datasets may not be suitable for the DBH extraction techniques due to a lack of points in this region. The accompanying supplemental material includes a 0.25 ha (50 m × 50 m) UAS SfM-derived point cloud from Swayze et al. [18] acquired at 65 m altitude above ground level at 90% front and side image overlap, using a crosshatch flight pattern and nadir camera orientation.

**Fig. 2:**
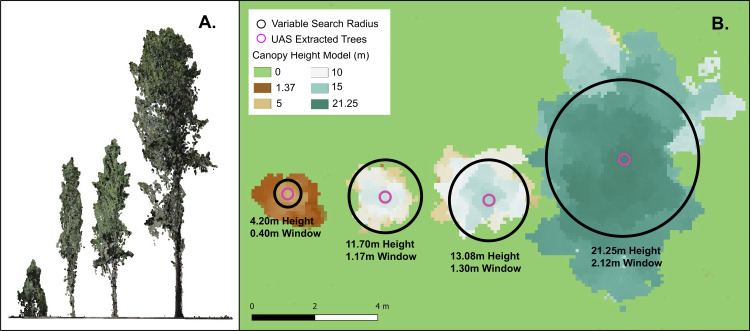
Example of the variable search window for tree extraction being scaled for different tree heights in the supplemental dataset.

**Fig. 3:**
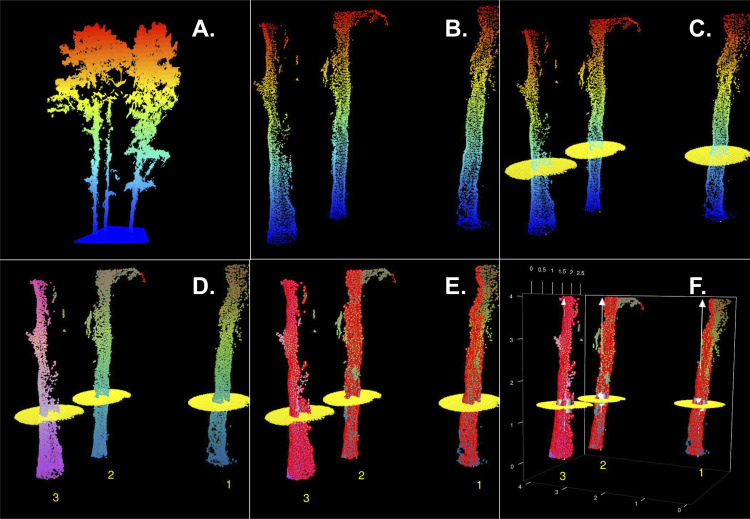
Progression of DBH extraction steps including (A) an example of the normalized point cloud, (B) the subset stem section from 0.1 to 4.0 m above ground, (C) the identified candidate tree locations, (D) classified tree points with tree IDs, (E) classified stem points, and (F) extracted DBH inventory.

UAS-SfM point clouds underwent a series of steps to prepare them for tree metric extraction, including 1) ground classification, 2) height normalization (Fig. 2A and Fig. 3A), and 3) canopy height model (CHM) rasterization. First, the SfM point cloud was imported into RStudio statistical software 4.0.4 [23] using the lidR Package [24]. Next, ground points were classified using a cloth simulation filter approach outlined in Zhang et al. [25], with a class threshold of 20 cm, a cloth resolution of 20 cm, medium rigidness, 500 iterations for simulating the cloth, and a stock time step setting of 0.65. Following ground classification, the point cloud was height normalized (i.e., replacing the elevation of each point with its height above the ground classified point surface) using a spatial interpolation k-nearest neighbor approach. The interpolator used an inverse-distance weighting of 10 nearest neighbors, using a power of 2 for inverse distance weighting and a maximum radius of 50 cm. The normalized point clouds were rasterized to a 10 cm spatial resolution CHM using a point to raster algorithm, which uses the highest point found in a pixel to assign the height value.

### Tree height and DBH extraction

Following the generation of 10 cm CHMs, extraction of individual tree locations and heights was completed using a local maximum variable window function from the R ForestTools package [26]. The function was parameterized based on research conducted in similar forest systems [16], [17], and only detected trees greater than 1.37 m tall. The function utilized a moving window search algorithm with a variable radius (Equation 1) to analyze CHMs identify local maximum pixels as single tree locations (Fig. 2B). Based on the height of each pixel, the variable window radius (meters) is adjusted based on the local maximum pixel height (meters) by a factor of 0.1. The outputs from this function return a list of single tree locations (x and y) and heights estimated in meters.

(1) variablewindowradius=localmaximumpixelx0.1

After individual tree height extraction, the point cloud is processed to extract DBH measurements using the R TreeLS package [27]. DBH extraction happens through five interconnected steps: (1) point cloud filtering, (2) tree mapping, (3) tree ID attribution, (4) stem point classification, and (5) DBH detection (Fig. 3). First, points below 0.1 m and above 4.0 m were removed from the normalized point cloud to focus on points representing the stem segment for DBH characterization (Fig. 3B) and substantially reducing processing time. Second, the treeMap function identifies candidate tree stem locations using a hough transform circle search algorithm (Fig. 3C). The algorithm detected the radius and circle center coordinates of potential tree locations by analyzing a two-dimensional slice of the point cloud between 1.32 and 1.42 m above ground. The tree mapping algorithm was parameterized to analyze points with a minimum point density of 0.001 points per square meter and a maximum potential stem diameter of 1 m. Third, the treePoints function crops points within a 0.5 m radius of each tree map coordinates and assigns a unique tree ID (Fig. 3D). Fourth, points with tree ID features are filtered for outliers and classified as stem points using the stemPoints function (Fig. 3E). The outliers are identified by applying a hough transform circle search noise filtering algorithm with a maximum stem diameter of 1.5 m and a minimum point density of 0.1 points per square meter. Points belonging to each tree ID are iteratively fit by the hough transform circle algorithm in 0.5 m vertical segments; points falling too far outside of the circle fit to each stem segment are removed from the resulting point cloud. Finally, each assigned tree ID iteratively feeds into the tlsInventory function to estimate the tree's DBH. The function operates by extracting a slice of the classified stem points that is 0.1 m thick and centered at 1.37 m, setting the height of all extracted points to 1.37 m, rasterizing the points, and fitting a circle to the raster through least-squares estimation using a random sample consensus approach. The resulting output of these steps is a file with stem locations (x and y) and stem radius estimated in centimeters (Fig. 3F). All point cloud processing and tree metric extraction steps are demonstrated in Supplemental Script Part 1 for use in the R environment.

### Matching, filtering, and modelling of missing DBH values

**Fig. 4:**
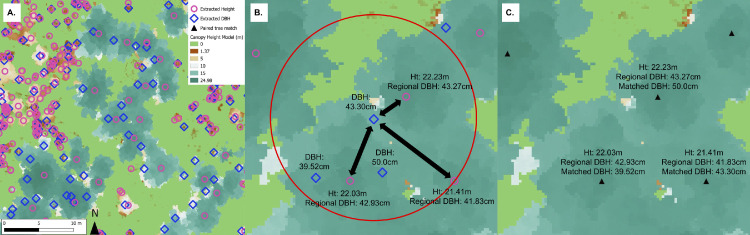
Progression of extracted tree height and DBH dataset matching process, including (A) an example canopy height model with extracted tree height (fuchsia) and DBH locations (blue), (B) 4 m buffer (shown in red) identifying three candidate extracted tree heights for a targeted DBH value, and (C) the final paired height and DBH values (shown as black triangles).

Extraction of tree metrics from the point cloud resulted in two datasets, each containing coordinates for either the tree heights or DBHs (Fig. 4A). Although the DBH extraction process provides reasonable estimates for many trees within the dataset, it is known that the precision of DBH values extracted from point clouds can vary widely based on the removal of outliers from the circle fitting process and issues during SfM image alignment. Additionally, past work has shown these approaches can range in the percentage of tree DBHs successfully extracted between 10 and 80% [28], [29]. To tie these metrics together, both datasets went through a multistep spatial matching process to: (1) predict DBH from a regional function for all extracted tree heights, (2) spatially match extracted DBHs with tree heights, (3) filter matched tree values using the regional height to DBH relationship, and (4) predict missing DBH values using matched UAS tree values. These steps are demonstrated in Supplemental Script Part 2 for use in the R environment.

**Fig. 5:**
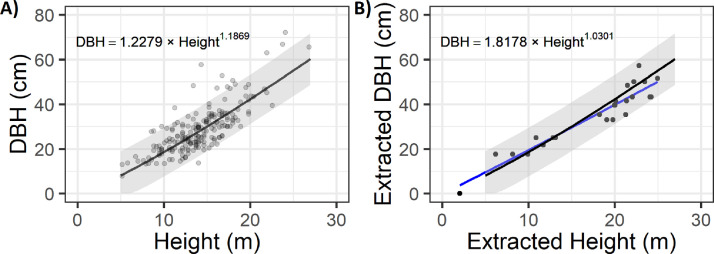
Height to DBH predictive model from (A) regional FIA data and (B) UAS-extracted and matched data. The regional model is always shown in black and UAS model in blue.

First, to aid in filtering any possible outlier DBH values, regional forest inventory data is used to develop a height to DBH relationship to filter matched UAS DBH and height values. The original implementation of this method drew upon the United States Forest Inventory and Analysis (FIA) program [30] to create this relationship. Regional FIA data were filtered to contain plots with a site index within ±2 m of the study site and with >70% of the basal area belonging to the study site's dominant species (ponderosa pine). The remaining data were used to generate a regional model using a power function to predict DBH as a function of height (Fig. 5A) using the R nlme package [31]. This regional function predicted DBH for each of the extracted tree heights for use as a filter while matching the extracted height and DBH values. The regional inventory dataset from Swayze et al. [18] is available as a Supplemental file.

Second, an extracted tree DBH was targeted and matched with extracted heights and their corresponding regionally predicted DBH values within a 4 m radius (Fig. 4B). The absolute difference between the extracted DBH and the regionally predicted DBHs was determined for all candidate heights. The pair with the smallest difference was considered the correct match and removed from further consideration (Fig. 4C). This process continued until all extracted tree DBHs had been iteratively matched with the extracted height that minimized the regional DBH difference.

**Fig. 6:**
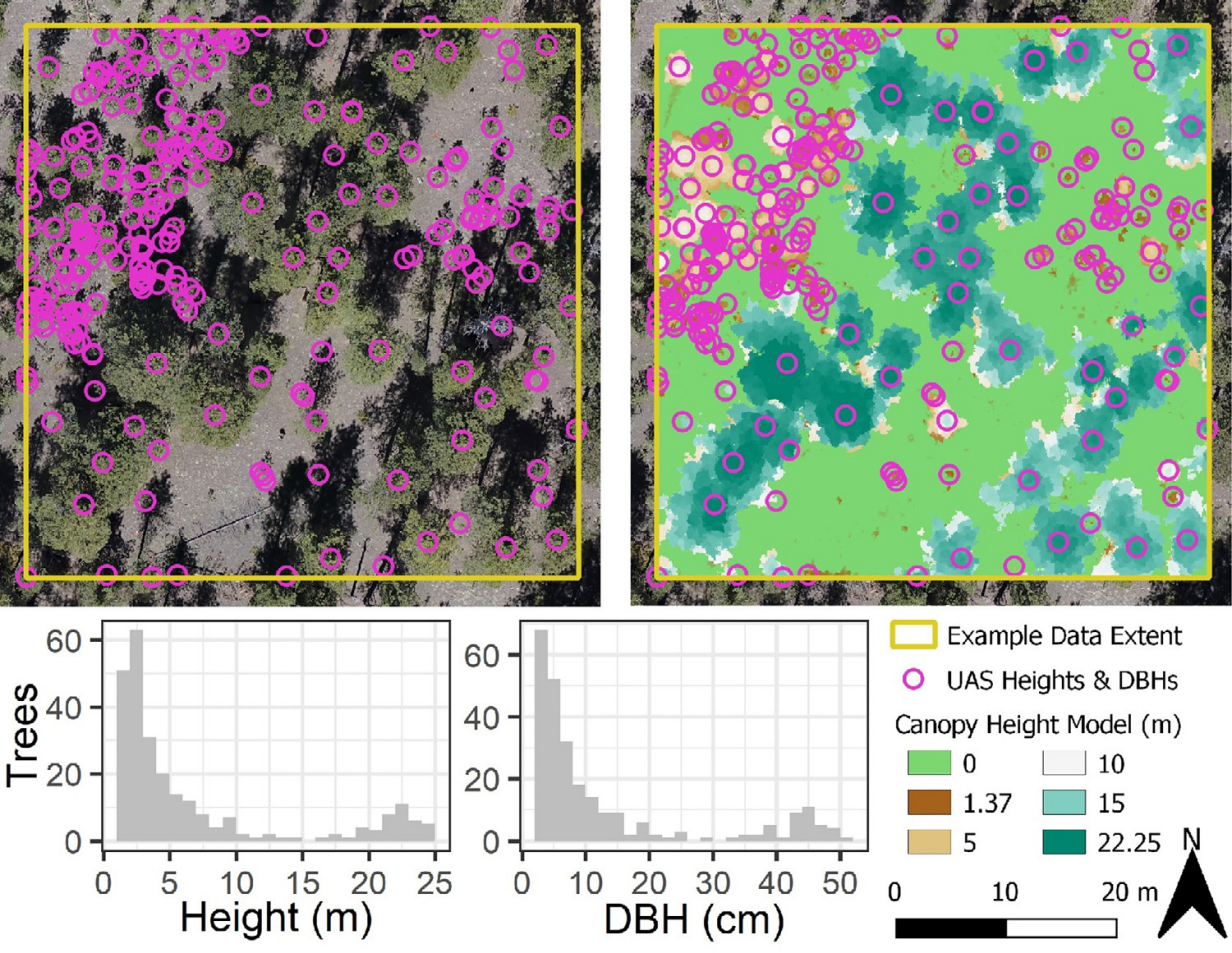
Maps of supplemental UAS dataset including orthophoto and canopy height model overlaid with UAS extracted tree locations and summarized histograms of extracted tree heights and UAS modeled DBHs.

Third, the matched height and DBH values were filtered to remove DBHs outside of the 90^th^ percentile bounds of the regional tree height to DBH relationship (Fig. 5B). Filtering against the regional prediction bounds helped remove any outlier DBH values that might impact subsequent modeling of missing DBHs. Fourth, a UAS-based height to DBH regression using a power function was created using the successfully matched and filtered tree values. The UAS model was used to predict missing DBH values for unmatched tree heights, resulting in a complete dataset of extracted tree locations with heights and DBHs (Fig. 6).

## Method validation

This method was initially presented and validated by Swayze et al. [18] using an intensively stem mapped site of nearly 900 trees. The validation augments what is presented here by spatially matching the UAS extracted and matched trees with field stem mapped trees characterized for height and DBH using traditional field methods. In total, validation used 30 UAS datasets collected at a range of altitudes, camera angles, and flight patterns. This analysis revealed that tree extraction accuracy was maximized at over 90% of trees for nadir camera angles using a crosshatch flight design. The extracted tree heights had errors smaller than traditional field inventory techniques. While off-nadir camera angles and crosshatch flight patterns increased the rate of DBH extraction to >40%, filtering with regional inventory data during the matching process removed an average of 60% of the extracted DBH values as they created a height to DBH relationship that did not conform to the regional height to DBH model. However, the filtering process caused the R^2^ values between UAS and stem mapped DBH values to exceed 0.75, significantly improving previous methods [19,29].

Within Swayze et al. [18], prediction of DBH from the UAS extracted and matched height and DBH values provided a strong approximation of the stem mapped distribution of DBH values. The greatest DBH errors were noted for the smallest diameter trees, which were generally underrepresented in the UAS extracted DBH data, leading to overestimation of these DBHs. The study also points out how other UAS extracted tree metrics like crown area or local stem density might improve the representation of the smallest diameter trees when modeling DBH. Following the modeling of missing DBH values, Swayze et al. [18] summarized plot-level estimates of trees per hectare and basal area per hectare with correlations typically exceeding 0.8 for most of the 30 tested UAS acquisitions. The best performing acquisitions characterized the study site's basal area within 5% of the stem mapped value, with off-nadir or crosshatch flight patterns maximizing precision.

## Appendix Supplementary materials

Image, application 1

Image, application 2

Image, application 3

Image, application 4
